# HupB Is a Bacterial Nucleoid-Associated Protein with an Indispensable Eukaryotic-Like Tail

**DOI:** 10.1128/mBio.01272-17

**Published:** 2017-11-07

**Authors:** Joanna Hołówka, Damian Trojanowski, Katarzyna Ginda, Bartosz Wojtaś, Bartłomiej Gielniewski, Dagmara Jakimowicz, Jolanta Zakrzewska-Czerwińska

**Affiliations:** a Laboratory of Molecular Biology of Microorganisms, Hirszfeld Institute of Immunology and Experimental Therapy, Polish Academy of Sciences, Wrocław, Poland; b Department of Molecular Microbiology, Faculty of Biotechnology, University of Wrocław, Wrocław, Poland; c Department of Biochemistry, University of Oxford, Oxford, United Kingdom; d Laboratory of Molecular Neurobiology, Neurobiology Center, Nencki Institute of Experimental Biology, Polish Academy of Sciences, Warsaw, Poland; University of Hyderabad; Harvard School of Public Health

**Keywords:** HU, HupB, *Mycobacterium*, chromosome dynamics, chromosome organization, nucleoid-associated proteins, NAPs

## Abstract

In bacteria, chromosomal DNA must be efficiently compacted to fit inside the small cell compartment while remaining available for the proteins involved in replication, segregation, and transcription. Among the nucleoid-associated proteins (NAPs) responsible for maintaining this highly organized and yet dynamic chromosome structure, the HU protein is one of the most conserved and highly abundant. HupB, a homologue of HU, was recently identified in mycobacteria. This intriguing mycobacterial NAP is composed of two domains: an N-terminal domain that resembles bacterial HU, and a long and distinctive C-terminal domain that contains several PAKK/KAAK motifs, which are characteristic of the H1/H5 family of eukaryotic histones. In this study, we analyzed the *in vivo* binding of HupB on the chromosome scale. By using PALM (photoactivated localization microscopy) and ChIP-Seq (chromatin immunoprecipitation followed by deep sequencing), we observed that the C-terminal domain is indispensable for the association of HupB with the nucleoid. Strikingly, the *in vivo* binding of HupB displayed a bias from the origin (*oriC*) to the terminus (*ter*) of the mycobacterial chromosome (numbers of binding sites decreased toward *ter*). We hypothesized that this binding mode reflects a role for HupB in organizing newly replicated *oriC* regions. Thus, HupB may be involved in coordinating replication with chromosome segregation.

## IMPORTANCE

We currently know little about the organization of the mycobacterial chromosome and its dynamics during the cell cycle. Among the mycobacterial nucleoid-associated proteins (NAPs) responsible for chromosome organization and dynamics, HupB is one of the most intriguing. It contains a long and distinctive C-terminal domain that harbors several PAKK/KAAK motifs, which are characteristic of the eukaryotic histone H1/H5 proteins. The HupB protein is also known to be crucial for the survival of tubercle bacilli during infection. Here, we provide *in vivo* experimental evidence showing that the C-terminal domain of HupB is crucial for its DNA binding. Our results suggest that HupB may be involved in organizing newly replicated regions and could help coordinate chromosome replication with segregation. Given that tuberculosis (TB) remains a serious worldwide health problem (10.4 million new TB cases were diagnosed in 2015, according to WHO) and new multidrug-resistant *Mycobacterium tuberculosis* strains are continually emerging, further studies of the biological function of HupB are needed to determine if this protein could be a prospect for novel antimicrobial drug development.

## INTRODUCTION

Bacterial chromosomal DNA must be efficiently compacted (in *Escherichia coli*, ~1,000× compaction) to fit inside the small cell compartment (1, 2), but it must also be available for the protein machineries involved in various cellular processes, such as DNA replication, chromosome segregation, transcription, and translation. In contrast to the situation in eukaryotic organisms, these processes occur simultaneously in bacteria. Thus, the bacterial chromosome (called the nucleoid) undergoes dynamic changes during the cell cycle (3–7). Bacteria lack histones; instead, the dynamic organization of the chromosome is maintained (to a large extent) by nucleoid-associated proteins (NAPs) (8–13). The NAPs are the most abundant proteins associated with the bacterial chromosome, and their cellular levels change during the growth cycle. Thus far, the most extensively investigated NAPs are those from *Escherichia coli* (14–17). These small, basic proteins have been shown to compact DNA into independent topological regions of ~10 kb, called microdomains (7), by bridging DNA (i.e., H-NS) or by bending/wrapping DNA around themselves (e.g., HU, IHF, Fis, Dps) (8, 18). In addition to their involvement in chromosomal organization, NAPs are involved in other cellular processes, such as DNA replication (e.g., HU, IHF, Fis) (19, 20), recombination and DNA repair (HU) (21), and global transcriptional regulation (H-NS, IHF, HU) (9, 22, 23).

HU is one of the most conserved and abundant NAPs in bacteria (14). In *E. coli*, HU exists as a dimer of closely related alpha- and beta-chains that share 70% identity at the amino acid sequence level (24, 25). HU binds DNA as a homo- or heterodimer, depending on the growth phase: HUαα predominates in the exponential phase, while HUαβ predominates in the stationary phase (26). These forms of *E. coli* HU do not exhibit sequence specificity, but they do prefer AT-rich sequences and/or distorted DNA (11, 27). Interestingly, the HU isoforms display different DNA-binding affinities and thus may modulate global nucleoid organization during *E. coli* growth. In addition to its role in chromosome compaction, HU contributes to initiation of replication by stabilizing the prereplication complex (19), and it can modulate transcriptional regulation (23).

The *Mycobacterium* genus encompasses both pathogenic species (e.g., *M. tuberculosis* and *M. leprae*, which cause tuberculosis and leprosy, respectively) and saprophytic species (e.g., *M. smegmatis*). The members of this genus are aerobic, rod-shaped, Gram-positive bacteria that have a thick mycolic acid-containing cell wall that protects them from hydrophilic substances, including many antibiotics. Recent studies have revealed that mycobacteria exhibit an unusual mode of cell elongation and division (28, 29). In contrast to other rod-shaped bacteria, such as *E. coli* and *Bacillus subtilis*, *Mycobacterium* incorporates newly synthesized peptidoglycan apically and often divides asymmetrically to generate two unevenly sized daughter cells. The existing studies of the mycobacterial cell cycle have mainly focused on chromosome replication and segregation (30–33), meaning that little is known about the architecture of the mycobacterial chromosome and its dynamics during the cell cycle. Given that tuberculosis (TB) remains a serious worldwide health problem, with 10.4 million new TB cases diagnosed in 2015 according to WHO (34), and that new multidrug-resistant *M. tuberculosis* strains are currently emerging (35), the mycobacterial cell cycle should be studied in the hopes of identifying new drug targets.

Many mycobacterial NAPs have only recently begun to be identified, due to their relative lack of sequence homology to their *E. coli* counterparts. These include Dps (36), NapM (37), and HupB (called also Hlp) (38), which is arguably the most intriguing mycobacterial NAP. Unlike *E. coli* HU, HupB has two domains: an N-terminal domain that resembles bacterial HU (~40% identity to *E. coli* HU) and a long distinctive C-terminal domain (CTD) that is present exclusively in mycobacteria and other *Actinobacteria* (39). The long C-terminal extension contains several PAKK/KAAK motifs, which are characteristic of members of the eukaryotic histone H1/H5 protein family. HupB (22 kDa; pI 12.5) binds DNA in a sequence-independent manner. However, similar to *E. coli* HU, it exhibits a preference toward AT-rich regions and particular DNA structures, such as Holliday junctions and replication forks (39, 40). Deletion of *hupB* is not lethal for *M. smegmatis* or *M. tuberculosis*, but strains lacking HupB are reportedly more sensitive to stress conditions, such as cold shock, UV radiation, and isoniazid treatment (*M. smegmatis*) (41), or are unable to proliferate in macrophages (*M. tuberculosis*) (42).

HupB has been suggested as a potential target for the development of therapies against tuberculosis (43), and it is regarded as a major mycobacterial NAP. However, no study has investigated its *in vivo* binding to DNA on the chromosome scale. Here, we describe the function of HupB in chromosome organization and demonstrate that the long C-terminal extension of HupB is indispensable for its *in vivo* association with the mycobacterial nucleoid.

## RESULTS

### The binding pattern of HupB reflects the global organization of the *M. smegmatis* chromosome.

**FIG 1:**
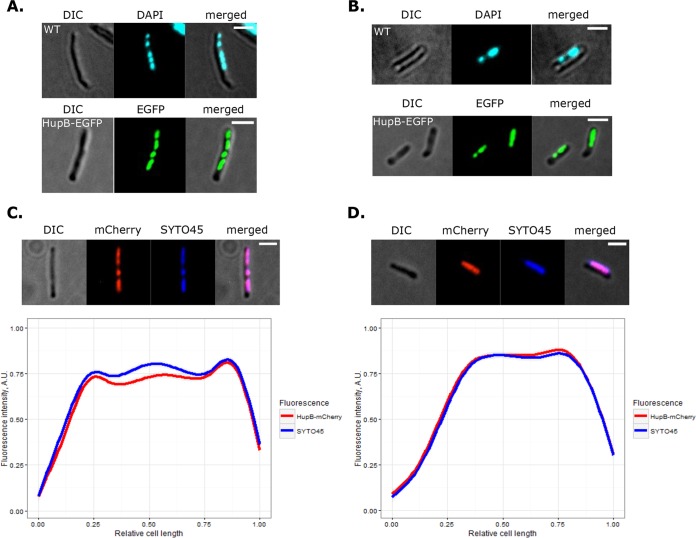
Nucleoid staining of HupB-EGFP and HupB-mCherry strains. (A and B) DAPI staining of WT and HupB-EGFP cells from log phase and stationary phase, respectively. (C and D, top panels) SYTO 45 staining of HupB-mCherry cells from log- and stationary-phase cultures, respectively. (Bottom panels) Fluorescence profiles along the long cell axis, as measured from the distant cell pole of SYTO 45-stained HupB-mCherry cells from log-phase (*n* = 50) and stationary-phase (*n* = 50) cultures, respectively. Scale bar, 2 μm.

In *E. coli*, HU shows low-specificity binding along the chromosome, yielding a dispersed fluorescent signal of HU-fluorescent protein (FP) throughout the nucleoid (10). HupB was previously localized in *M. smegmatis* by immunostaining using a mouse anti-histone H1 antibody, which was chosen based on the resemblance of the C-terminal domain of HupB (HupB_CTD_) to members of the eukaryotic H1/H5 histone family (44). The previous study suggested that HupB only partially occupies the nucleoid and that the average number of molecules per cell (117 in log phase) is substantially lower than that in *E. coli*. To comprehensively evaluate the subcellular localization of HupB, we constructed strains in which HupB fused with either enhanced green fluorescent protein (EGFP) or red (mCherry) FPs was expressed from its native promoter at the endogenous chromosomal locus (for details, see Text S1 in the supplemental material). HupB-EGFP (48 kDa) or HupB-mCherry (47 kDa) protein bands of the expected size were observed in extracts of fusion protein-expressing cells (Fig. S1). The colony morphologies (data not shown) and growth rates of HupB-EGFP and HupB-mCherry strains were similar to those of the wild-type (WT) strain (Fig. S1). Fluorescence microscopy revealed that both HupB-EGFP and HupB-mCherry fusion proteins were usually visible as a compact cluster(s) of discrete and bright foci of various sizes within each cell, reflecting HupB-EGFP-DNA macrocomplexes (Fig. 1). Further analysis revealed that the fluorescent patterns of these HupB-EGFP-DNA macrocomplexes resembled the nucleoids observed in 4′,6-diamidino-2-phenylindole (DAPI)-stained WT cells (Fig. 1A and B), during both the log and the stationary phases. Since DAPI tends to additionally condense the nucleoid, we compared the compaction (as a percentage of cell length) of SYTO 45-stained nucleoids in WT cells with the shape delineated by the fluorescent foci in HupB-EGFP cells. Both fluorescence focus patterns and the degree of nucleoid condensation (75% ± 9% of cell length in HupB-EGFP cells, versus 83% ± 8% in WT cells [mean ± standard deviation]; *n* = 94) were similar in analyzed cells, prompting us to conclude that HupB occupies the entire nucleoid. To confirm this, we stained HupB-mCherry cells with SYTO 45, measured the fluorescence intensity profiles along the long cell axis in relation to the distant cell pole in cells from the log and stationary phases and generated averages for the signal distributions inside the cell (Fig. 1C and D). The fluorescence profiles of mCherry and SYTO 45 did indeed overlap in the analyzed strains, confirming that HupB-FP colocalizes with the nucleoid during both the log phase and the stationary phase, when the chromosome is globally more condensed.

10.1128/mBio.01272-17.1 TEXT S1  Additional details regarding our materials and methods. Download TEXT S1, DOCX file, 0.03 MB. Copyright © 2017 Hołówka et al. 2017 Hołówka et al. This content is distributed under the terms of the Creative Commons Attribution 4.0 International license.

10.1128/mBio.01272-17.2 FIG S1  Characteristics of the constructed fluorescent reporter strains. (A) Growth curves of the analyzed strains (adjusted via loess model; R software with ggplot2 package [72]). (B) Expression of fusion proteins of the expected sizes confirmed by Western blotting. Green triangle indicates HupB-EGFP or HupB_ΔCTD_-EGFP fusion protein. Red triangle indicates HupB-mCherry, HupB-PAmCherry or HupB_ΔCTD_-PAmCherry. The wild-type strain (WT) served as a negative control. Download FIG S1, PDF file, 0.04 MB. Copyright © 2017 Hołówka et al. 2017 Hołówka et al. This content is distributed under the terms of the Creative Commons Attribution 4.0 International license.

We next examined the numbers of HupB-EGFP monomers per cell by performing Western blotting of total *M. smegmatis* cell proteins with antibodies raised against EGFP, using purified recombinant EGFP (rEGFP; Cell Biolabs) to generate a standard curve. We found that, similar to *E. coli* HU, HupB was highly abundant in log-phase cells (30,000 to 60,000 HupB-EGFP molecules/cell, compared to 20,000 to 50,000 *E. coli* HU molecules/cell [14]).

**FIG 2:**
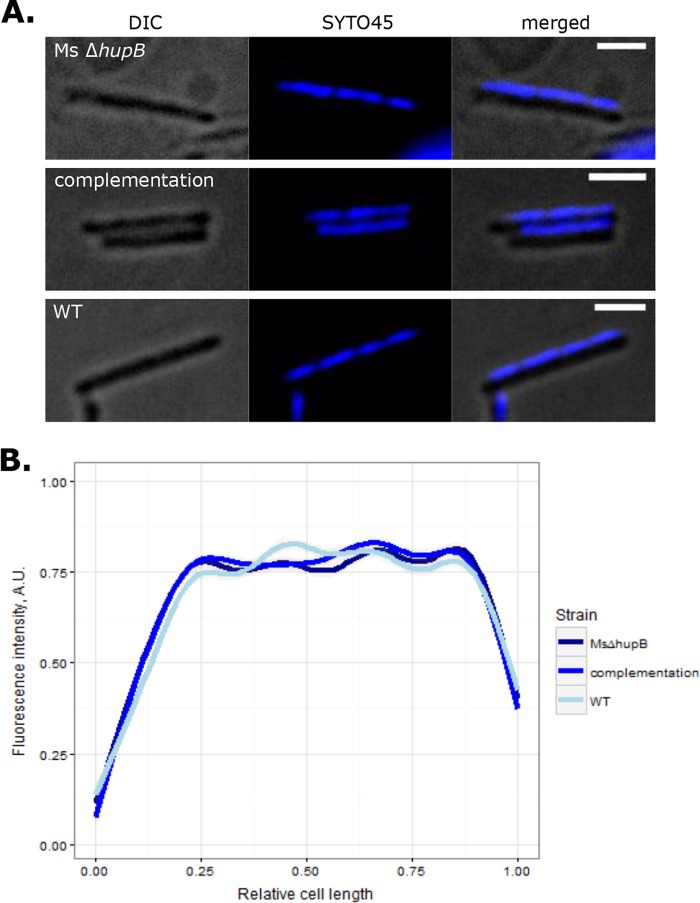
Influence of *hupB* deletion on chromosome condensation. (A) Micrographs showing representative SYTO 45-stained cells of the Δ*hupB*, complementation, and WT strains. (B) Fluorescence profiles along the long cell axis of analyzed strains (*n* = 45), as measured from the distant cell pole. Scale bar, 2 μm.

As a structural NAP, HupB is expected to influence chromosome organization in mycobacteria. To determine whether it is involved in the chromosome compaction of *M. smegmatis*, we constructed a *hupB* deletion strain. Microscopic analysis of SYTO 45-stained Δ*hupB* cells revealed that the distribution of nucleoid inside the cell was similar to the distribution found in WT cells and the complementation strain (Fig. 2). Moreover, the analyzed strains exhibited comparable degrees of chromosome compaction (~80 to 90% of the cell length). These observations suggest that HupB may be replaced by other NAP(s), such as the recently described mIHF (45) or NapM (37), and/or that HupB is specific to local-level changes in chromosome organization that were not detected by our experimental strategy.

**FIG 3:**
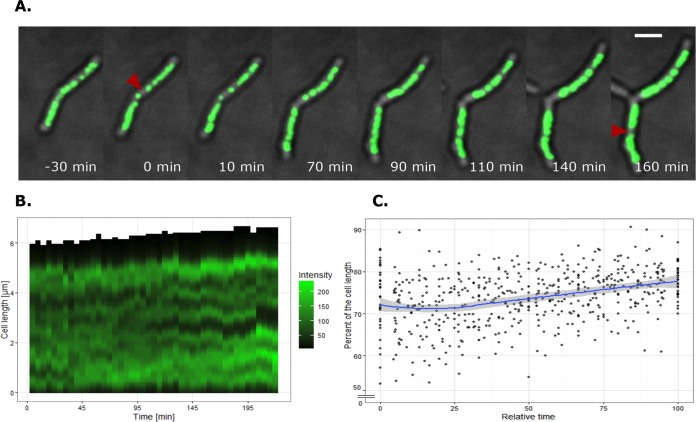
Real-time visualization of HupB-EGFP-DNA macrocomplexes. (A) Time-lapse analysis of sister chromosome separation during the cell cycle. *t*0 indicates sister chromosome separation, and a red triangle marks each separation event. Scale bar, 2 μm. (B) Graph presenting the real-time localizations of HupB-EGFP-DNA macrocomplexes in a representative cell. The fluorescence intensity along the long cell axis was measured from the old cell pole. Here, *t*0 does not correspond to sister chromosome separation but rather to the middle of the cell cycle. (C) Chart showing real-time mean chromosome condensation levels with corresponding 95% confidence intervals. Chromosome condensation is shown as a percentage of cell length and was measured between two sister chromosome separation events (*n* = 30).

To investigate the dynamic localization of HupB-FP-DNA macrocomplexes, we performed time-lapse fluorescence microscopy (TLFM) of HupB-EGFP cells. Our TLFM analysis revealed that the HupB-EGFP-DNA complexes did not occupy fixed positions inside the cell, but rather were constantly diverging and merging together (Fig. 3A and B; Movie S1 and S2). Prior to cell division, HupB-EGFP-DNA complexes were arranged in two distinct and compact clusters that reflected the separation of sister chromosomes (Fig. 3A, red triangles; Movie S1, red arrow). Despite the dynamic localization of HupB-EGFP-DNA complexes, the global nucleoid condensation remained similar (70 to 80% of the cell length) (Fig. 3C). Hence, the local dynamic behavior of HupB-EGFP-DNA complexes presumably reflects the constant changes of nucleoid organization that occur during the cell cycle, such as those induced by ongoing chromosome replication, the segregation of newly replicated chromosomal regions, and transcription.

10.1128/mBio.01272-17.8 MOVIE S1  Time-lapse imaging of HupB-EGFP cells on agar plates (IBIDI). Fluorescent spots represent DNA-HupB-EGFP complexes. Red arrows indicate the sister chromosome separation events. Images were acquired automatically every 10 min. Scale bar, 2 μm. Download MOVIE S1, AVI file, 0.1 MB. Copyright © 2017 Hołówka et al. 2017 Hołówka et al. This content is distributed under the terms of the Creative Commons Attribution 4.0 International license.

10.1128/mBio.01272-17.9 MOVIE S2  Time-lapse imaging of HupB-EGFP strain obtained with the ONIX system. Fluorescent spots represent DNA-HupB-EGFP complexes. Red arrows indicate the sister chromosome separation events. Images were acquired automatically every 5 min. Scale bar, 2 μm. Download MOVIE S2, AVI file, 0.3 MB. Copyright © 2017 Hołówka et al. 2017 Hołówka et al. This content is distributed under the terms of the Creative Commons Attribution 4.0 International license.

Taken together, our results show that the characteristic pattern of HupB-FP foci and their dynamic localization reflects the real-time organization of the *M. smegmatis* chromosome. This confirms that HupB-FP may be used to study the chromosome dynamics of this organism during the cell cycle.

### The C-terminal domain is indispensable for the biological function of HupB in *Mycobacteria*.

The mycobacterial HU (HupB) and those of other *Actinobacteria* have an additional CTD that makes them unique among the NAPs. We thus set out to examine the biological function of the CTD. The truncated form of HupB lacking the CTD (HupB_ΔCTD_; 11 kDa) was previously found to have a tertiary structure, similar to that of the canonical HUs from *E. coli* and *Bacillus stearothermophilus* (46, 47), but it exhibited a lower *in vitro* DNA binding affinity and different substrate specificity from WT HupB (39, 40). Here, to establish the role of the CTD in chromosome organization and dynamics *in vivo*, we constructed a strain in which the 3′-terminal region of *hupB*, encoding HupB_CTD_, was deleted (*ΔhupB*_CTD_). The growth rate of *ΔhupB*_CTD_ cells in rich medium showed no significant difference from WT cells and the complementation strain (Fig. S2A). However, similar to the previously reported *hupB* deletion strain (41), HupB_ΔCTD_-producing cells were more susceptible to isoniazid than WT *M. smegmatis* (~90% growth inhibition versus the WT under 1.5 μg/ml of isoniazid [Fig. S2B]). Since isoniazid is activated by a product of the *katG* gene, we hypothesized that *katG* gene expression can be altered by the disturbed interaction between HupB_ΔCTD_ and the DNA, which is consistent with previous reports (48, 49).

10.1128/mBio.01272-17.3 FIG S2  Growth curves of *M. smegmatis* ΔhupB and HupB_ΔCTD_ strains in comparison to the WT and complementation strain. Measurements were carried out under optimal conditions (A) and in the presence of 1.5 mg/liter isoniazid (B) (growth curves were adjusted by loess model; R software with ggplot2 package [72]). Download FIG S2, PDF file, 0.03 MB. Copyright © 2017 Hołówka et al. 2017 Hołówka et al. This content is distributed under the terms of the Creative Commons Attribution 4.0 International license.

**FIG 4:**
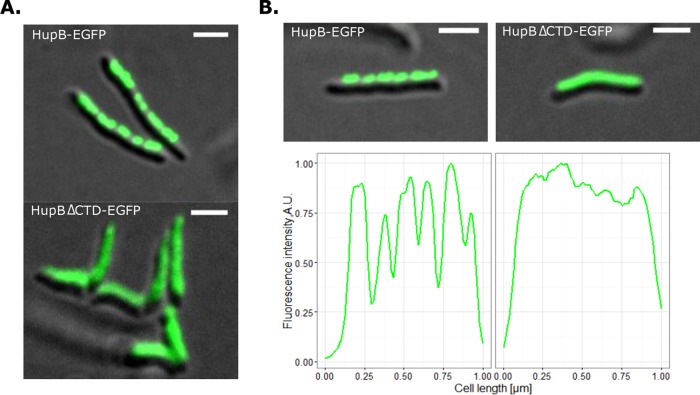
Subcellular localizations of HupB_ΔCTD_-EGFP and HupB-EGFP. (A) Micrographs showing representative cells of the analyzed strains. (B) EGFP fluorescence profiles of representative HupB-EGFP and HupB_ΔCTD_-EGFP cells along the long cell axis, as measured from the distant cell pole. Scale bar, 2 μm.

To analyze the subcellular localization of HupB_ΔCTD_, we constructed an *M. smegmatis* strain that produced HupB_ΔCTD_ fused with EGFP. Interestingly, microscopic analysis revealed a dispersed fluorescence signal in these cells (Fig. 4), with no evidence of the clusters of discrete fluorescent foci seen in the HupB-EGFP strain. This suggests that, unlike HupB-EGFP, HupB_ΔCTD_-EGFP is not associated with the nucleoid.

**FIG 5:**
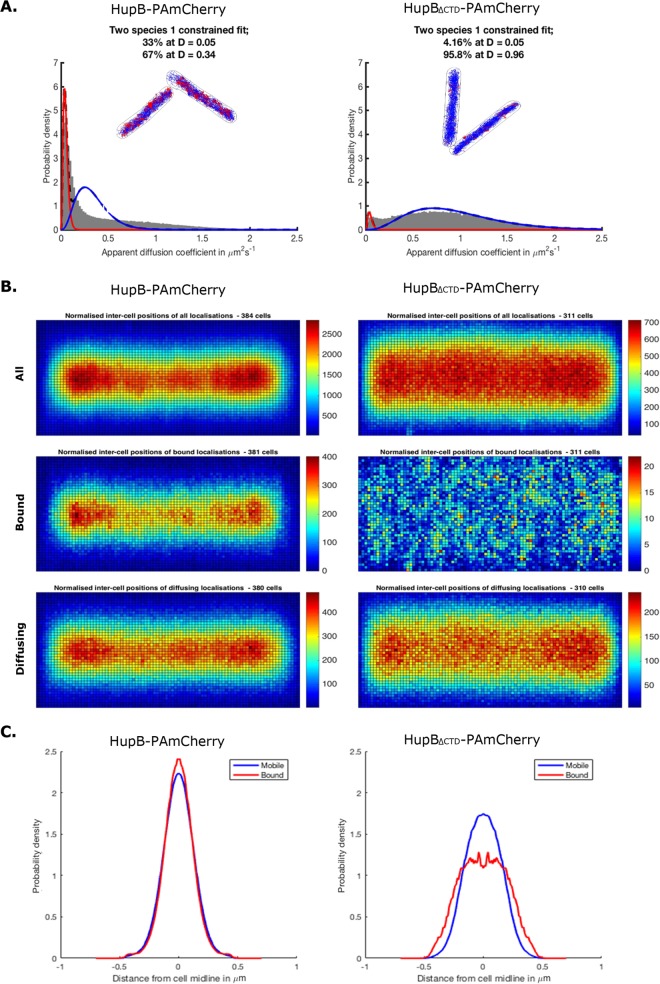
PALM analysis of the mobility and subcellular localization of HupB-PAmCherry and HupB_ΔCTD_-PAmCherry fusion proteins. (A) Histograms of the apparent diffusion coefficients with visualization of the tracks taken by the immobile (red) and diffusing (blue) particle fractions of HupB-PAmCherry and HupB_ΔCTD_-PAmCherry cells (*n* = 384 and *n* = 311, respectively). (B) Diagrams presenting the normalized intercellular positions of HupB-PAmCherry and HupB_ΔCTD_-PAmCherry particles. (C) Histograms showing the distributions of HupB-PAmCherry and HupB_ΔCTD_-PAmCherry particles in the cell cross-section.

The above findings led us to propose that the CTD is crucial for the ability of HupB to bind DNA *in vivo*. To test this hypothesis, we used photoactivated localization microscopy (PALM) (50), which can be applied to visualize single protein molecules and track the mobility changes that may be caused by binding to the nucleoid. We constructed *M. smegmatis* strains in which the *hupB* gene had been deleted at the endogenous locus and were complemented with *hupB*_ΔCTD_ or *hupB*, both of which were fused with the PA*mcherry* gene, which encodes the photoactivatable mCherry protein (HupB_ΔCTD_-PAmCherry and HupB-PAmCherry, respectively). The presence of the fusion proteins in cell extracts was confirmed by Western blotting (Fig. S1), and the growth rates (Fig. S1) and colony morphologies (data not shown) of HupB-PAmCherry and HupB_ΔCTD_-PAmCherry cells were similar to those of WT cells. Analysis of particle mobility revealed that the ratio of diffusing versus immobile particles was considerably higher for HupB_ΔCTD_-PAmCherry cells than for HupB-PAmCherry cells (approximately 20:1 versus 2:1, respectively) (Fig. 5A), suggesting that a substantial fraction of the truncated protein may be present in the cytoplasm rather than bound to the nucleoid.

Diagrams presenting normalized intracell positions of the analyzed proteins showed that the diffusing and immobile particles of HupB-PAmCherry were both localized centrally along the long axis of the cell (Fig. 5B). Interestingly, the highest concentration of HupB-PAmCherry particles was observed in the cell quarters (20 to 25% and 75 to 80% of the total cell length) and thus resembled the positioning of ParB/*oriC* complexes (31–33, 51) in exponentially growing cells. In contrast to HupB-PAmCherry, both fractions of HupB_ΔCTD_-PAmCherry were more dispersed inside the cell (Fig. 5B). Differences in the subcellular localizations of HupB-PAmCherry and HupB_ΔCTD_-PAmCherry were also clearly seen on histograms presenting the distribution of analyzed particles in cell cross-sections (Fig. 5C, left and right histograms, respectively).

These results were consistent with those obtained from our microscopic studies (Fig. 4) and supported the hypothesis that truncated HupB lacking the CTD does not associate with the nucleoid. The high proportion of mobile particles suggests that HupB_ΔCTD_-PAmCherry cannot effectively bind DNA and/or that HupB_ΔCTD_-DNA complexes are not stable. Previous *in vitro* studies showed that the N- and C-terminal domains of HupB act synergistically in DNA binding and that CTD-deleted HupB shows a significantly lower DNA binding affinity (39, 40). Our results expand upon this by showing that the C-terminal domain of HupB is crucial for stable DNA binding *in vivo*.

### HupB binding displays a bias against the terminus of the *M. smegmatis* chromosome.

**FIG 6:**
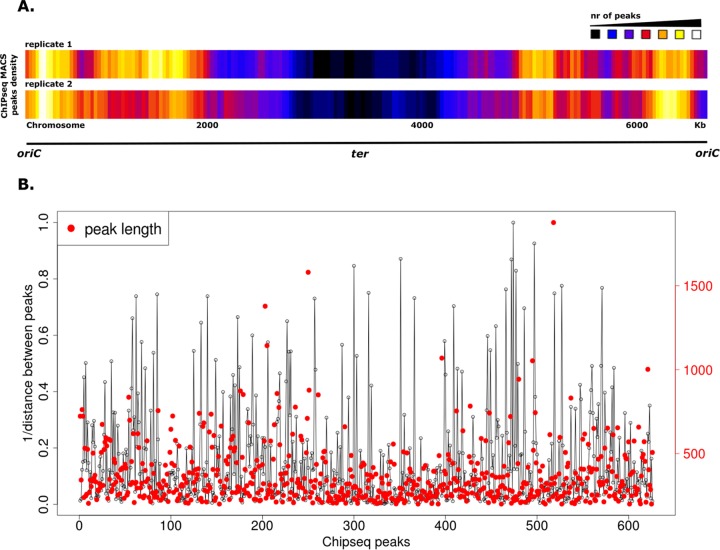
Analysis of HupB binding sites on the *M. smegmatis* chromosome. (A) Heat maps of the distributions of HupB-FLAG binding sites in two biological replicates. (B) Graph showing the density of HupB-FLAG binding sites on the chromosome. The *x* axis represents all ChIP-Seq peaks (the red dot indicates the length of a given peak [in base pairs]; the red scale on *y* axis on the right). The straight lines on the *y* axis indicate the inverse of the distance between two given ChIP-Seq peaks (i.e., the longer the line, the closer the peaks).

Our comprehensive microscopic analyses revealed that, in contrast to the *E. coli* HU (10), HupB-FP was seen as discrete fluorescent foci within each cell (Fig. 1 and 3A). Moreover, the high-resolution microscopic findings suggested that HupB is concentrated near cellular positions of newly replicated *oriC* regions (Fig. 5B) (31–33, 51). To examine whether HupB binds to specific chromosomal regions, we performed chromatin immunoprecipitation followed by deep sequencing (ChIP-Seq) (52) and then sought to verify the binding mode of CTD-deleted HupB. We performed ChIP-Seq experiments using *M. smegmatis* strains producing HupB or HupB_ΔCTD_ proteins fused with three repeats of the FLAG epitope (FLAG_3_) (53). The presence of the fusion proteins in cell extracts was confirmed by Western blotting (Fig. S3), and HupB-FLAG_3_ and HupB_ΔCTD_-FLAG_3_ strains exhibited growth rates (Fig. S3) and colony morphology (data not shown) similar to those of the WT strain. HupB-FLAG_3_-DNA and HupB_ΔCTD_-FLAG_3_-DNA nucleoprotein complexes fixed with formaldehyde in the log phase of growth were immunoprecipitated using magnetic beads coated with anti-FLAG monoclonal mouse antibody (see Materials and Methods). The immunoprecipitated DNA was then isolated and amplified, and the generated library of DNA fragments was subjected to deep sequencing. To exclude unspecific interactions with magnetic beads, we used a WT strain lacking the FLAG epitope as a negative control for our ChIP-Seq experiments. Enriched regions (indicating HupB binding sites along the chromosome) were determined by comparison to the background noise level, which was estimated versus the input DNA of each ChIP-Seq replicate. Using the data obtained from this analysis, we established chromosomal binding maps for the analyzed proteins. Consistent with the findings of our microscopic analyses, the ChIP-Seq data obtained with HupB_ΔCTD_-FLAG_3_ cells showed little enrichment in either of the biological replicates compared to the input DNA (Fig. S4). In contrast, we identified numerous HupB binding sites along the whole chromosome (Fig. 6A).

10.1128/mBio.01272-17.4 FIG S3  Characteristics of constructed FLAG-tagged strains. (A) Growth curves of the analyzed strains (adjusted by loess model; R software with ggplot2 package [72]). (B) Expression of the HupB-FLAG (top panel) and the HupB_ΔCTD_-FLAG (bottom panel) fusion proteins confirmed by Western blotting. The wild-type strain (WT) served as a negative control. Download FIG S3, PDF file, 0.3 MB. Copyright © 2017 Hołówka et al. 2017 Hołówka et al. This content is distributed under the terms of the Creative Commons Attribution 4.0 International license.

10.1128/mBio.01272-17.5 FIG S4  Comparison of enriched regions on the *M. smegmatis* chromosome for every ChIP-Seq replicate. Enriched regions (dark blue) of biological replicates of HupB-FLAG (A and B), HupB_ΔCTD_-FLAG (C and D), and the WT (E and F) were determined by comparison to the background noise level (light blue), which was estimated versus the input DNA of each ChIP-Seq replicate. Download FIG S4, PDF file, 0.6 MB. Copyright © 2017 Hołówka et al. 2017 Hołówka et al. This content is distributed under the terms of the Creative Commons Attribution 4.0 International license.

Interestingly, the HupB-FLAG binding sites were distributed unevenly along the *M. smegmatis* chromosome (Fig. 6A): most of the ChIP-Seq peaks were located around *oriC*, and their number decreased toward the chromosomal terminus (*ter*). This asymmetry of the binding profile was observed in both biological replicates performed using HupB-FLAG_3_. The results of ChIP-Seq were normalized using input DNA isolated from the cells at the same growth phase (see Materials and Methods), thereby eliminating the replication-associated gene dosage effect that can be seen during the log phase of growth. Hence, we believe that the obtained pattern shows that HupB binds asymmetrically on the chromosome.

The identified HupB-FLAG binding sites included 626 ChIP-Seq peaks that were confirmed in two biological replicates and absent in the WT (Fig. S5A). Given our finding that the HupB level reaches up to 30,000 dimers per cell during log phase, the protein should bind every 230 bp on the *M. smegmatis* chromosome. Instead, our ChIP-Seq peaks suggested that the binding sites were dispersed at ~11,000-bp intervals. This presumably indicates that HupB can form higher oligomers bringing together relatively distant DNA regions. Such a binding mode would explain the presence of long ChIP-Seq peaks (1,000 bp or longer) (Fig. 6B; the red dots and scale in the figure indicate the ChIP-Seq peak lengths) distributed along the entire chromosome. Interestingly, our analysis of the ChIP-Seq peak distribution also revealed that the HupB binding sites formed irregular clusters on the chromosome (Fig. 6B).

10.1128/mBio.01272-17.6 FIG S5  Analysis of binding sites identified from the ChIP-seq experiments. (A) Peaks overlap between HupB-FLAG replicates (HupB1 and HupB2) and the WT replicates (WT1 and WT2). (B and C) Chromosomal binding map of HupB-FLAG (B) in comparison to the WT negative control (C). Download FIG S5, PDF file, 0.3 MB. Copyright © 2017 Hołówka et al. 2017 Hołówka et al. This content is distributed under the terms of the Creative Commons Attribution 4.0 International license.

Similar to other NAPs and aside from its structural role in chromosome organization, the HupB protein can influence gene expression (49, 54). From among the 626 ChIP-Seq peaks identified for HupB-FLAG, we selected the 350 ChIP-Seq peaks that showed the highest enrichments (≥5-fold) in both biological replicates and categorized them based on their location with respect to the beginning of a given open reading frame (see Materials and Methods) (Fig. S5B and C). Although HupB seemed to prefer AT-rich regions (39, 40), which are more frequent within promoters, most of the binding sites were identified within gene bodies (~66% of all ChIP-Seq peaks) (Fig. S5B). This might suggest that HupB indirectly regulates gene expression, as seen for the global gene regulation observed for other NAPs. Therefore, the binding of HupB within a certain gene would be expected to downregulate that gene and/or affect the expression of neighboring genes by introducing topological changes in the DNA. Moreover, HupB oligomerization may lead to the formation of loops that join relatively distant DNA regions and/or trigger the downregulation of certain genes. This could explain HupB-mediated regulation of the *katG* gene (48, 49).

Taken together, the data from our ChIP-Seq analysis were consistent with our PALM results and revealed that HupB is asymmetrically distributed on the *M. smegmatis* chromosome, with most of the reads localized around *oriC*. In contrast, we were unable to identify discrete binding sites for CTD-deleted HupB. These findings suggest that although HupB_ΔCTD_ resembles the canonical HU protein from *E. coli*, CTD-deleted HupB loses its DNA binding affinity and/or forms a less stable complex with DNA.

## DISCUSSION

Mycobacterial HupB is unique among the bacterial HU proteins. It possesses an additional, long CTD that occurs exclusively in *Actinobacteria* (Fig. S6) and contains several PAKK/KAAK repeats, which are characteristic of the eukaryotic histone H1/H5 proteins (39). These basic C-terminal motifs have also been identified in other DNA binding proteins of *Actinobacteria*, including Ku (55) and topoisomerase I (56, 67). In *Streptomyces coelicolor*, two HU-like proteins are involved in chromosome organization: HupA compacts chromosomes in vegetative hyphae, while HupS is involved in chromosome condensation during sporulation (57). In *Mycobacterium leprae*, HupB (called Lpb or Hlp) appears to be responsible for adhesion to host respiratory epithelial cells (58). Deletion of the *hupB* gene in *Mycobacterium* spp. is not lethal, but rather it affects growth under stress conditions (41, 42). While the biochemistry of the HupB protein has been relatively well studied, its role in chromosome organization *in vivo* is poorly understood. In this study, we analyzed the *in vivo* binding of *M. smegmatis* HupB on a chromosome scale and investigated the biological function of its C-terminal domain.

10.1128/mBio.01272-17.7 FIG S6  Multiple-sequence alignment of the C-terminal domain of HupB homologues in *Actinobacteria*. Basic amino acid residues are marked in blue. Download FIG S6, PDF file, 0.1 MB. Copyright © 2017 Hołówka et al. 2017 Hołówka et al. This content is distributed under the terms of the Creative Commons Attribution 4.0 International license.

### The binding pattern of HupB reflects the dynamic nature of the *M. smegmatis* chromosome.

Microscopic analysis of HupB fluorescent reporter strains revealed that the HupB-FP forms compact clusters of bright fluorescent foci within the cell. HupB-FP was found to colocalize with the DAPI/SYTO 45-stained *M. smegmatis* chromosome (Fig. 1), both in exponentially growing cells and during the stationary phase, when the global condensation of the nucleoid is substantially higher. Interestingly, cells lacking HupB protein did not exhibit any significant difference in global nucleoid organization (Fig. 2), suggesting that any involvement of HupB in chromosome compaction occurs on a local scale. This finding is similar to previously reported chromosome conformation capture (Hi-C) experiments in *C. crescentus*, which demonstrated that deletion of the *hu1* and *hu2* genes, which encode the HU1 and HU2 proteins, significantly decreased short-range interactions but did not affect global chromosome organization (59). Alternatively, the global function of HupB may be replaced by those of other NAPs, such as mIHF (45) or NapM (37).

Since the pattern of HupB-FP foci reflected the organization of the nucleoid *in vivo*, we used the HupB-EGFP strain to analyze the dynamics of the mycobacterial chromosome during the cell cycle. Previously, HU-FP strains have been used to examine the real-time chromosome organizations of model organisms, such as *E. coli* (60) and *C. crescentus* (61). Here, our time-lapse experiments revealed that the HupB-EGFP-DNA macrocomplexes exhibited dynamic changes during the cell cycle (Fig. 3B), even though overall chromosome condensation did not change (70 to 80% of the cell length) (Fig. 3C). The constant diverging and merging of macrocomplexes (Movie S1 and S2) presumably reflect the dynamic changes of the chromosome during ongoing replication, the segregation of newly replicated chromosomal regions, and gene transcription.

In contrast to the *E. coli* HU, the HupB-FP-DNA macrocomplexes were found to localize as discrete but evenly distributed fluorescent foci. This prompted us to question whether HupB could occupy certain chromosomal regions. Indeed, PALM analysis of the mobility and distribution of HupB-PAmCherry particles showed that a relatively high fraction of immobile molecules (33%) (Fig. 5A) were localized centrally along the cell’s long axis (Fig. 5B, middle diagram). This population presumably corresponds to DNA-associated HupB-PAmCherry particles. Interestingly, these immobile HupB-PAmCherry particles seemed to be unevenly distributed, exhibiting their highest densities in the cell quarters (Fig. 5B, middle diagram). This localization pattern resembles that of ParB complexes in exponentially growing *M. smegmatis* cells (31–33, 51). ParB, which is a component of the ParAB*S* segregation system in mycobacteria, binds *parS* sequences localized near *oriC* to create large nucleoprotein complexes called segrosomes (30). Thus, HupB may be recruited to the *oriC-*proximal region of newly replicated chromosomes to compact them and facilitate their segregation, thereby acting as a small-scale chromosome organizer. The remaining fraction of diffusing particles (67%) (Fig. 5A) exhibited dynamic but cell-centered localization (Fig. 5B, bottom diagram), perhaps reflecting transient DNA binding events. The distribution profiles of the diffusing and immobile particles in the cell cross-section corroborated this observation (Fig. 5C). Notably, the area with the highest density of HupB-PAmCherry particles (Fig. 5B, top diagram) corresponded to the subcellular localization of HupB-FP-DNA macrocomplexes obtained using traditional fluorescence microscopy (Fig. 1 and 3A).

**FIG 7:**
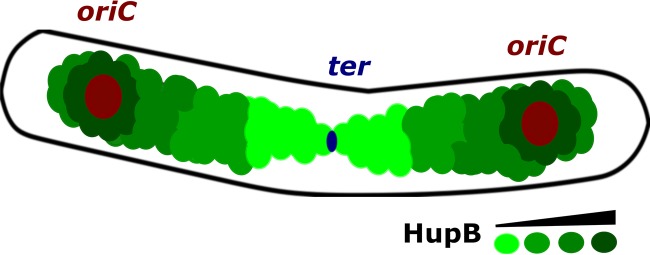
Distribution of HupB on chromosomes in exponentially growing *M. smegmatis* cells. An increasing number of HupB binding sites is indicated by the green color scale. The positions of the newly replicated *oriC*s in the cell quarters were consistent with those found in our previous studies (32, 33, 51). The scheme is based on the results obtained from our PALM analysis (Fig. 5) and ChIP-Seq (Fig. 6) experiments.

To further investigate whether HupB binds specific chromosome regions, we performed ChIP-Seq using FLAG-tagged HupB. From our results, we generated a global binding map of HupB (Fig. 6A), which indicated the arrangement of HupB binding sites on the *M. smegmatis* chromosome. The ChIP-Seq peaks were clustered around *oriC* and decreased toward *ter*. This binding pattern of HupB was consistent with the PALM results (Fig. 5B). Similar asymmetries in chromosomal binding sites have been reported for other DNA binding proteins, including SeqA (62), Noc (63), and the recently described *C. crescentus* GapR (64). In the binding model proposed for GapR (64), the passage of a replication fork drives the dissociation of GapR from DNA, resulting in GapR-mediated cell cycle regulation. In the case of HupB, the asymmetrical binding mode could reflect a functional role in the organization of the newly replicated *oriC* regions (Fig. 7). Future work is needed to test this hypothesis.

Similar to the HU proteins of other bacteria, *M. smegmatis* HupB binds DNA with no sequence specificity, showing only preferences for particular DNA structures and AT-rich regions (39, 40). In *E. coli*, HU binding sites occur at regular intervals along the chromosome, resulting in dispersed fluorescence with HU-FP (10). In contrast, we identified relatively few HupB binding sites (*n* = 626), given the size of the *M. smegmatis* chromosome (~7 Mbp) and the amount of the protein found in log-phase cells (up to 30,000 dimers per cell). Such a scattered binding pattern may suggest that HupB creates higher oligomers. These may result in the bending and/or looping of longer DNA segments. Recent studies showed that *E. coli* HU can multimerize to create relatively long, inflexible DNA filaments (65). This could be another model for HupB oligomerization. Such a binding mode of HupB could explain why we obtained many long reads (1,000 bp or longer) in our ChIP-Seq analysis. Interestingly, most of the identified binding sites (~66% of all ChIP-Seq peaks) (Fig. S5B) were located inside genes, suggesting that HupB plays an architectural role, such as through relatively longer-range DNA interactions, or/and indirectly regulates the expression of neighboring genes by changing DNA topology.

Taken together, our data suggest that HupB binds at distinct chromosomal loci distributed along the entire *M. smegmatis* chromosome. Thus, DNA-HupB-FP macrocomplexes may be visualized as discrete, bright fluorescent foci inside the cell. Their dynamic behavior reflects the constant changes experienced by the chromosome during the cell cycle and is consistent with the recent observation that HupB and topoisomerase A (TopA) interact to limit the relaxing activity of TopA (66). This interplay between TopA and HupB would contribute to maintaining the homeostasis of chromosome topology. Moreover, our observation that there is a substantially larger amount of HupB during the exponential growth phase than had been previously reported (44) suggests that this protein (similarly to HU from *E. coli*) plays a crucial role in the chromosome organization of actively replicating cells. Interestingly, our high-resolution microscopic analysis suggested that HupB may be preferentially recruited to *oriC*-proximal regions, where it presumably contributes to their organization. This decrease in the density of HupB binding sites from *oriC* to *ter* was also observed in our ChIP-Seq experiments. Thus, the local-scale binding mode of HupB harmonizes with the dynamic behavior of the chromosome during the exponential growth phase.

### The C-terminal domain of HupB is indispensable for effective *in vivo* DNA binding.

Finally, we examined the biological function of the unique C-terminal domain of HupB. Previous *in vitro* studies had shown that CTD-deleted HupB exhibited a significantly decreased DNA binding affinity and that both domains of HupB act synergistically in DNA binding (39, 40). To elucidate the *in vivo* role of the HupB CTD, we prepared fluorescent reporter strains that produced CTD-deleted HupB fused with fluorescent protein (HupB_ΔCTD_-FP). We then analyzed the subcellular localization of the fusion protein, performed high-resolution microscopic experiments, and produced a global chromosome binding map of HupB_ΔCTD_ by using ChIP-Seq.

HupB_ΔCTD_ cells showed no significant difference in their growth rate under optimal conditions compared to WT cells (Fig. S2A). However, similar to the HupB deletion mutant (41), HupB_ΔCTD_ cells were susceptible to a relatively low concentration of isoniazid (Fig. S2B). Since HupB may regulate the expression of the *katG* gene, the product of which activates isoniazid, we hypothesized that the DNA binding of HupB_ΔCTD_ may be disturbed. Fluorescence microscopy revealed that the HupB_ΔCTD_-EGFP fusion protein did not appear to associate with the nucleoid, as it showed a dispersed fluorescence signal (Fig. 4), without the clusters of bright fluorescent foci that were characteristic of HupB-EGFP (Fig. 1A and B). This observation is consistent with the findings of previous *in vitro* studies (39, 40) and the results of our PALM experiments. The CTD-deleted protein had a higher fraction (~96%) of diffusing particles than did HupB-PAmCherry (67%), suggesting that the binding of HupB_ΔCTD_-PAmCherry to DNA is unstable (Fig. 5A). The lack of the C-terminal tail, which harbors basic amino acid repeats predicted to interact with the negatively charged DNA backbone, may decrease the DNA binding affinity of HupB_ΔCTD_ and/or the stability of the HupB_ΔCTD_-DNA complex. This is consistent with *in vitro* findings indicating that the DNA binding affinity of HupB_ΔCTD_ is lower than that of HupB (39, 40). Similarly, lysine repeats in the C-terminal domain of TopA enhance the stability of the enzyme-DNA complex and increase the processivity of the topoisomerase (67). Given the recent discovery that HupB undergoes posttranslational modifications, it seems likely that the DNA binding activity of the protein could be diminished by phosphorylation of the N-terminal threonine or by acetylation within the CTD (68, 69). By analogy, posttranslational modifications of linker histones H1/H5 occur within their C-terminal basic repeats, in addition to N-terminal domain modifications (70). Since a relatively high level of HupB is seen during the exponential growth phase (~30,000 dimers/cell), modifications of its long C-terminal tail may be the key mechanism for regulating HupB binding activity.

The binding defect of HupB_ΔCTD_ results in a more-dispersed distribution of HupB_ΔCTD_-PAmCherry particles along the cell compared to the native protein, which has a relatively high fraction of immobile, DNA-bound particles (Fig. 5A and B). In the cell cross-section, the immobilized HupB_ΔCTD_ particles were also dispersed (Fig. 5C), excluding their possible chromosomal localization. Additionally, our ChIP-Seq analysis failed to identify any binding site for HupB_ΔCTD_-FLAG. The fact that there was no enrichment along the *M. smegmatis* chromosome in comparison to the given input DNA confirmed our hypothesis that the binding of DNA by HupB_ΔCTD_ is disturbed.

In summary, our results indicate that the pattern of HupB-FP reflects the *in vivo* organization of the *M. smegmatis* chromosome. The HupB binding sites are arranged asymmetrically (Fig. 7), suggesting that HupB may help organize newly replicated *oriC* proximal regions and thus contribute to coordinating replication with chromosome segregation in actively dividing cells. The HupB C-terminal domain, which is unique among the bacterial HUs, seems to be indispensable for the *in vivo* binding of HupB. The lack of this CTD may destabilize HupB on the DNA strand and/or disturb the formation of the higher oligomers. Given that HupB is crucial for the survival of *M. tuberculosis* during infection, further studies into the biological functions of HupB, particularly the role of its C-terminal domain, may suggest directions for the development of novel antimicrobial drugs. Recently, the first attempt was made to inhibit *M. tuberculosis* HupB DNA binding activity using stilbene derivatives (43).

## MATERIALS AND METHODS

### DNA manipulations, bacterial strains, and culture conditions.

DNA manipulations were carried out using standard protocols (71). Reagents and enzymes were supplied by Thermo Scientific, Roth, and Sigma-Aldrich. Oligonucleotides were synthesized by Genomed or Sigma-Aldrich, and sequencing was performed by Genomed. All plasmids used to construct the *M. smegmatis* mc^2^ 155 mutant strains were propagated in the *E. coli* DH5α strain. *E. coli* cells were grown in LB broth or on LB agar plates (Difco) supplemented with the proper antibiotic(s) (ampicillin at 100 μg/ml, kanamycin at 50 μg/ml) and/or other compounds (5-bromo-4-chloro-3-indolyl-β-d-galactopyranoside [X-Gal] at 0.004%, isopropyl-β-d-1-thiogalactopyranoside [IPTG] at 0.5 mM), according to standard procedures. *M. smegmatis* mc^2^ 155 strains were grown either in 7H9 broth supplemented with 10% oleic acid-albumin-dextrose-catalase (OADC; BD) and 0.05% Tween 80 or on 7H10 agar plates (Difco) supplemented with 10% OADC, 0.5% glycerol, 0.004% X-Gal, and/or kanamycin (50 μg/ml) or 2% sucrose. Strains, plasmids, and oligonucleotides are listed in Table S1, and the construction of the *M. smegmatis* mc^2^ 155 mutant strains is described in Text S1.

10.1128/mBio.01272-17.10 TABLE S1  Oligonucleotides, plasmids, and *M. smegmatis* mc^2^ 155 strains used in the study. Download TABLE S1, DOCX file, 0.03 MB. Copyright © 2017 Hołówka et al. 2017 Hołówka et al. This content is distributed under the terms of the Creative Commons Attribution 4.0 International license.

### Fluorescent fusion protein quantification.

Quantification of HupB-EGFP fusion proteins was performed by Western blotting, with rEGFP (1 mg/ml; Cell Biolabs) used to generate a standard curve. *M. smegmatis* log-phase cells (OD_600_, 0.6 to 0.7) liquid cultures were collected, centrifuged (6,000 rpm, 5 min), resuspended in phosphate-buffered saline (PBS) with 1× sample buffer (Tris-HCl [pH 6.8] at 100 mM, glycerol at 20%, bromophenol blue at 0.2%, β-mercaptoethanol at 200 mM) and denatured at 95°C for 30 min. SDS-PAGE was performed according to standard procedures. After electrophoresis, the proteins were transferred to a nitrocellulose membrane (semidry transfer; Pierce G2 Fast Blotter; Thermo Scientific). Blots were blocked in Tris-buffered saline with Tween 20 with 5% milk and incubated with a primary monoclonal mouse anti-EGFP antibody (1:1,000; Sigma-Aldrich) followed by a horseradish peroxidase-conjugated secondary goat anti-mouse antibody (1:3,000; Santa Cruz Biotechnology). Band intensities were examined using Fiji software platforms (http://fiji.sc/Fiji). The amount of fluorescent fusion protein per cell was determined by standardization to CFU counts.

### Microscopy.

Snapshot imaging was performed using log-phase cells (OD_600_, 0.6 to 0.7) or stationary-phase cells (OD_600_, >2). *M. smegmatis* cultures were grown in liquid medium overnight, centrifuged (6,000 rpm, 5 min), resuspended in PBS, and smeared onto microscope slides. For visualization of the *M. smegmatis* chromosome, the cells were first incubated with DAPI (2 μg ml^−1^) for 2 h. For SYTO 45 staining, 200 μl of the culture was incubated for 15 min with SYTO 45 (0.25 μM) and then smeared onto microscopic slides. Dried samples were mounted with 5 μl of PBS-glycerol (1:1) solution and examined with a Zeiss Axio Imager Z1 fluorescence microscope equipped with a 100×  objective. Pictures were analyzed using the Fiji software and the R software (R Foundation for Statistical Computing, Vienna, Austria; https://www.r-project.org/), including the ggplot2 package (72).

### Time-lapse microscopy.

For real-time analysis, early log-phase *M. smegmatis* cultures (OD_600_, 0.2 to 0.4) grown in liquid medium were used. Experiments were performed by culturing cells either on an IBIDI μ-Dish (35 mm, low) with solid medium or in liquid medium using an ONIX microfluidic system. Images were recorded at 5- or 10-min intervals using a Delta Vision Elite inverted microscope equipped with a 100× oil immersion objective or with an inverted Zeiss Axio Observer fluorescence microscope equipped with a 100× oil immersion objective. Data were analyzed using the Fiji software and R software, including the ggplot2 package (72).

### PALM.

Single-molecule-tracking PALM was performed using a custom-built total internal reflection fluorescence microscope similar to a previously described setup (73). Photoactivatable mCherry (PAmCherry) was activated with a 405-nm laser, with excitation at 561 nm. For recording of bright-field cell images, an light-emitting diode (LED) source and condenser (ASI Imaging) were used. Molecule tracking and localization analysis were performed using custom-written MatLab software (MathWorks, Inc.). Bound and diffusing proteins were distinguished by calculating the apparent diffusion coefficient (D*), as follows: D* = MSD/(4Dt), where MSD is the mean squared displacement for each track with four steps. Due to cell confinement and motion blurring, D* is an apparent term (74).

### Chromatin immunoprecipitation.

ChIP was performed using log-phase (OD_600_, 0.6) liquid medium-grown cultures of *M. smegmatis* strains producing HupB-FLAG or HupB_NTD_-FLAG. As a negative control, WT log-phase culture was used. The cells were fixed with 1% formaldehyde for 30 min, the reaction was quenched with 150 mM glycine for 15 min, and then the cells were washed three times with cold PBS (54,000 rpm, 5 min, 4°C) and frozen at −80°C. To prepare lysates, pellets were resuspended in FA-1 buffer (HEPES-KOH at 50 mM [pH 7.5], NaCl at 140 mM, EDTA at 1 mM, Triton X-100 at 1%, and protease inhibitor cocktail [Thermo Scientific]), disintegrated with silica beads (0.1 mm) for 45 min, and sonicated on ice using 10 cycles of a 10-s pulse followed by a 50-s pause. The obtained lysates were centrifuged (5 min, 12,000 rpm, 4°C) and frozen at −80°C in 5% glycerol. For immunoprecipitation, 200 μg of total protein was incubated on a rotary shaker for 4 h at 4°C with a 15-μl packed-gel volume of anti-FLAG M2 magnetic beads (Sigma-Aldrich) and then washed twice with FA-1 buffer. Samples were processed in a final volume of 0.5 ml in two biological replicates, with input DNA controls (200 μg of total protein alone) included for each replicate. Samples were washed using a magnetic separator with sequential applications of FA-1 buffer, FA-2 buffer (HEPES-KOH at 50 mM [pH 7.5], NaCl at 500 mM, EDTA at 1 mM, Triton X-100 at 1%, and protease inhibitor cocktail) and TE (Tris-HCl at 10 mM [pH 8.0], EDTA at 1 mM). Immunoprecipitated samples were de-cross-linked overnight in TE containing 1% SDS at 65°C and then digested with proteinase K (final concentration, 0.05 mg/ml) for 1.5 h at 55°C. The immunoprecipitated DNA was extracted using phenol:chloroform:isoamyl alcohol (25:24:1) and precipitated with absolute ethanol.

### Library construction and Illumina sequencing.

The library of DNA fragments was prepared using a QIAseq Ultralow Input library kit (Qiagen). Briefly, the protocol includes DNA end repair, sequencing adapter ligation, cleanup, and PCR amplification. At the end of the procedure, quantification and quality evaluations were done using a Nanodrop spectrophotometer (Thermo Scientific), a Quantus fluorimeter (Promega), and a 2100 Bioanalyzer (Agilent). Second-generation sequencing was performed using a HiSeq 1500 sequencing platform (Illumina).

### Analysis of ChIP-Seq data.

The obtained FASTQ files were filtered according to read quality, and adapter sequences were trimmed using the Trimmomatic software (Usadel Lab; Aachen University, Aachen, Germany). The filtered FASTQ files were mapped to the genome of *M. smegmatis* strain mc^2^ 155 (from Ensembl bacteria release 35) using the Bwa aligner (Burrows-Wheeler aligner), and the Bwa mem algorithm was applied. The bam files were sorted and indexed. PCR duplicates were detected and removed by using the MarkDuplicates feature of Picardtools. The bam files for the ChIP and input samples were subjected to MACS analysis (MACS2 software) for ChIP-Seq peak detection. Peak calling was performed without building a model, using a shift size of 100 bp. The ChIP-Seq peaks were uploaded into the R environment as bed files, and the peaks were annotated to operons. Operon annotations were downloaded from http://operons.ibt.unam.mx/OperonPredictor/. A peak was defined as occurring in a promoter if it intersected a region between 150 bp upstream and 1 bp upstream of the start site of an operon/gene. Gene body peaks were annotated when a peak began and ended within a gene/operon body region. Peaks that did not fall into the promoter or gene body categories were defined as intergenic peaks. Peaks that fell into both the promoter and gene body categories were defined as mixed peaks. ChIP**-**Seq data are available upon request.
