# Supplementary material for: HupB Is a Bacterial Nucleoid-Associated Protein with an Indispensable Eukaryotic-Like Tail
Source: mBio. 2017 Nov 7;8(6):e01272-17. doi: 10.1128/mBio.01272-17 (PMC5676037; doi:10.1128/mBio.01272-17)
Supplement: TEXT S1 [file mbo006173577s1.docx]

**Text S1**

**Materials and Methods**

***M. smegmatis* mc^2^ 155 mutant strains construction**

The allelic replacement of gene encoding HupB (*Msmeg_2389*) protein with *hupB-egfp*/ *hupB-mcherry/hupB-flag* or *hupB_∆CTD_-egfp/hupB_∆CTD_-flag* was performed accordingly to Parish and Stoker (1). Flanking sequences containing the *Msmeg_2389* gene were amplified on a template of *M. smegmatis* mc^2^ 155 (WT) chromosomal DNA and using primer pairs, Ms_2388_Fw_HindIII/ Ms_2389_Rv_BamHI and Ms_HupB2_Fw_KpnI/ Ms_HupB2_Rv_PacI (Table S1). In case of *hupB_∆CTD_-egfp* flanking sequences were amplified using primer pairs, Ms_2388_Fw_HindIII/ Ms_2389_N_Rv_KpnI and Ms_HupB2_Fw_KpnI/ Ms_HupB2_Rv_PacI. The *egfp* gene with a short linker encoding 10 amino acid sequence at the 5ʹ-terminus (2) was PCR-amplified using primers GFP_BamHI_F_66 and GFP_KpnI_R_64 while three times repeated FLAG sequence with *BamH*I and *Kpn*I overhangs was prepared by hybridization of oligo pair FLAGx3_BamHI/KpnI_Fw and FLAGx3_BamHI/KpnI_Rv. PCR products were verified by sequencing and cloned into a p2NIL (kan*^R^*) vector. For construction of *M. smegmatis* mc^2^ 155 strain with deletion of the whole *hupB* gene or the 3’ – terminal sequence encoding CTD (92 – 209 aa) of HupB the analogous cloning strategy was used. Flanking regions were amplified on a template of *M. smegmatis* mc^2^ 155 (WT) chromosomal DNA using primer pairs Ms_2388_Fw_HindIII/ Ms_2389_5’_Rv_KpnI, Ms_HupB2_Fw_KpnI/ Ms_HupB2_Rv_PacI for *hupB* gene deletion and Ms_2388_Fw_HindIII/ Ms_2389_N_Rv_KpnI, Ms_HupB2_Fw_KpnI/ Ms_HupB2_Rv_PacI for deletion of 3’ – terminal sequence encoding CTD of HupB. PCR products were verified by sequencing and cloned into a p2NIL (kan*^R^*) vector. At the end, the pGoal17 cassette was cloned into the *Pac*I site of each p2NIL derivative.

*M. smegmatis* cells were then transformed with 200 ng of NaOH/EDTA-treated plasmid DNA (3) and unmarked mutants were selected accordingly to procedure described previously (1). DCO mutants were analyzed by PCR, Western blotting and/or DNA sequencing. Fluorescent proteins fusions were additionally verified by semi-native SDS-PAGE and subsequent detection with Pharos FX Plus Molecular Imager (4).

For construction of complementation strain and strains producing HupB-PAmcherry proteins, mycobacteriophage L5-based integration-proficient vector pMV306 (Kan^R^) was used (5). The pMV-HupB vector was obtained by amplification of *hupB* gene with the 200 bp upstream region on a template of *M. smegmatis* mc^2^ 155 (WT) chromosomal DNA using primers Ms_p_MS_hupB_Fw_HindIII/Ms_hupB_KpnI_Rv. PCR product was verified by sequencing and cloned into pMV306 vector. The *PAmcherry* gene (6) with a short linker encoding 11 amino acid sequence at the 5ʹ-terminus was PCR-amplified using primers PAmcherry_BamHI_Fw/ GFP_KpnI_R_64, verified by sequencing and cloned into p2NIL-HupB_∆CTD_ vector. Then, *EcoR*I/*Kpn*I fragment was subcloned from p2NIL-HupB_∆CTD_-PAmcherry vector to pMV306 resulting in construction of pMV-HupB_∆CTD_-PAmcherry vector. The pMV-HupB*-*PAmcherry vector was obtained by Gibson Assembly using starter pairs: KK_hupB_SalI_Fw/ KK_(paCh)_hupB_Rv and KK_(hupB)_paCh_Fw/ KK_SalI_paCh_Rv. *M. smegmatis* electrocompetent cells were transformed with pMV306 derivatives and recombinants were selected using kanamycin. Transformants were analyzed by PCR and/or semi-native SDS-PAGE and Western blotting. Growth rate of mutant strains was monitored with Bioscreen incubator for 48 h at different conditions.

**References**

1. Parish T, Stoker NG. 2000. Use of a flexible cassette method to generate a double unmarked Mycobacterium tuberculosis tlyA plcABC mutant by gene replacement. Microbiol Read Engl 146 ( Pt 8):1969–1975.

2. Ruban-Ośmiałowska B, Jakimowicz D, Smulczyk-Krawczyszyn A, Chater KF, Zakrzewska-Czerwińska J. 2006. Replisome localization in vegetative and aerial hyphae of Streptomyces coelicolor. J Bacteriol 188:7311–7316.

3. Hinds J, Mahenthiralingam E, Kempsell KE, Duncan K, Stokes RW, Parish T, Stoker NG. 1999. Enhanced gene replacement in mycobacteria. Microbiol Read Engl 145 ( Pt 3):519–527.

4. Jakimowicz D, Brzostek A, Rumijowska-Galewicz A, Zydek P, Dołzbłasz A, Smulczyk-Krawczyszyn A, Zimniak T, Wojtasz L, Zawilak-Pawlik A, Kois A, Dziadek J, Zakrzewska-Czerwińska J. 2007. Characterization of the mycobacterial chromosome segregation protein ParB and identification of its target in Mycobacterium smegmatis. Microbiol Read Engl 153:4050–4060.

5. Triccas JA, Parish T, Britton WJ, Gicquel B. 1998. An inducible expression system permitting the efficient purification of a recombinant antigen from Mycobacterium smegmatis. FEMS Microbiol Lett 167:151–156.

6. Subach FV, Patterson GH, Manley S, Gillette JM, Lippincott-Schwartz J, Verkhusha VV. 2009. Photoactivatable mCherry for high-resolution two-color fluorescence microscopy. Nat Methods 6:153–159.
