# Supplementary figures and images for: HupB Is a Bacterial Nucleoid-Associated Protein with an Indispensable Eukaryotic-Like Tail
Source: mBio. 2017 Nov 7;8(6):e01272-17. doi: 10.1128/mBio.01272-17 (PMC5676037; doi:10.1128/mBio.01272-17)

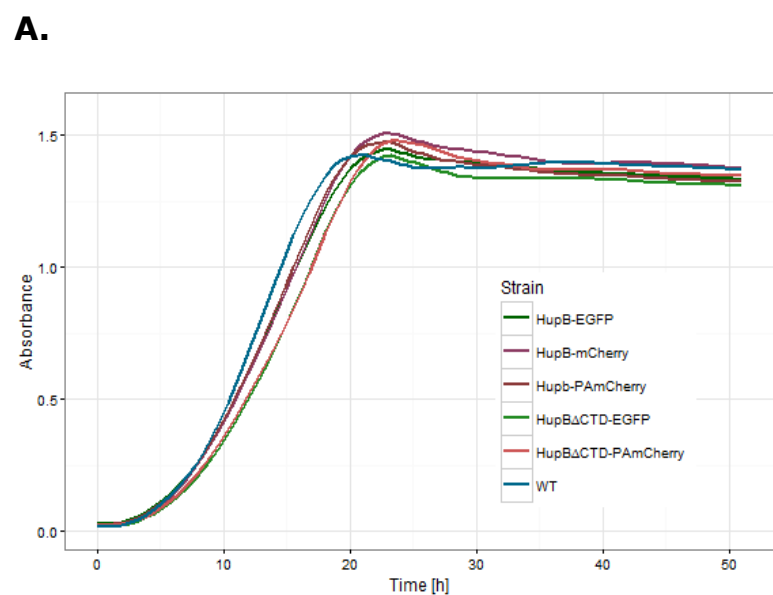

Fig. S1. Hołowka et al., 2017

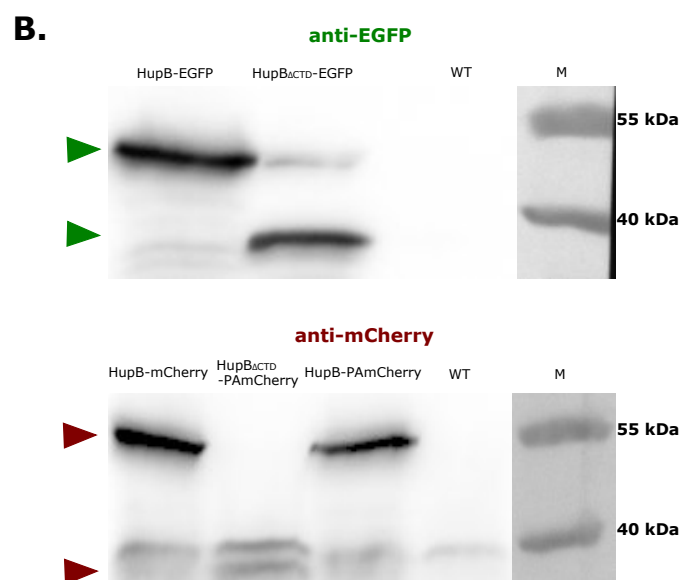

Supplement: FIG S1 [file mbo006173577sf1.pdf]

**A.**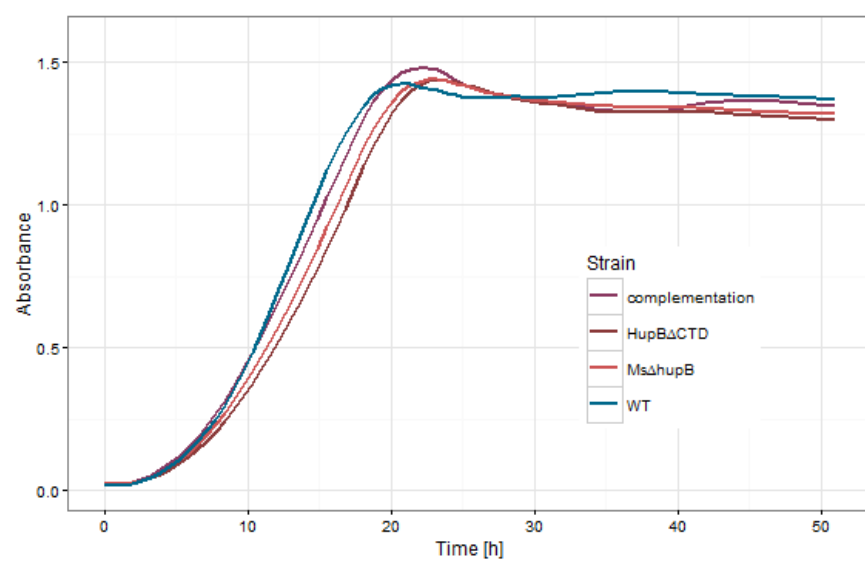**B.**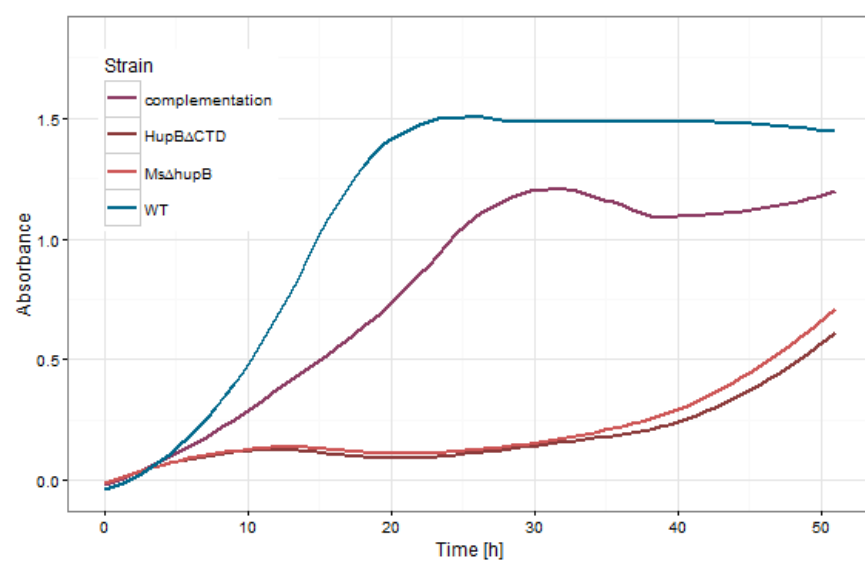

Fig. S2. Hołowka et al., 2017

Supplement: FIG S2 [file mbo006173577sf2.pdf]

**A.**

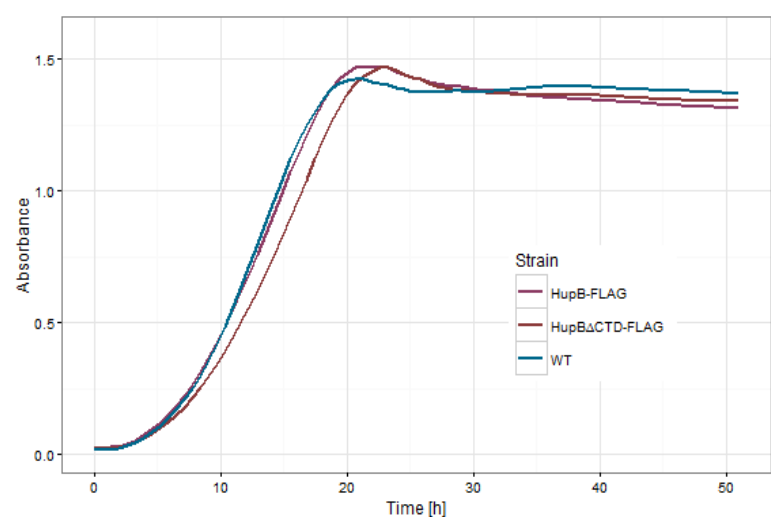

Fig. S3. Hołowka et al., 2017

**B.**

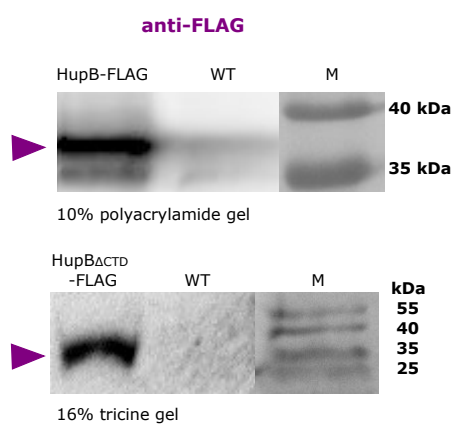

Supplement: FIG S3 [file mbo006173577sf3.pdf]

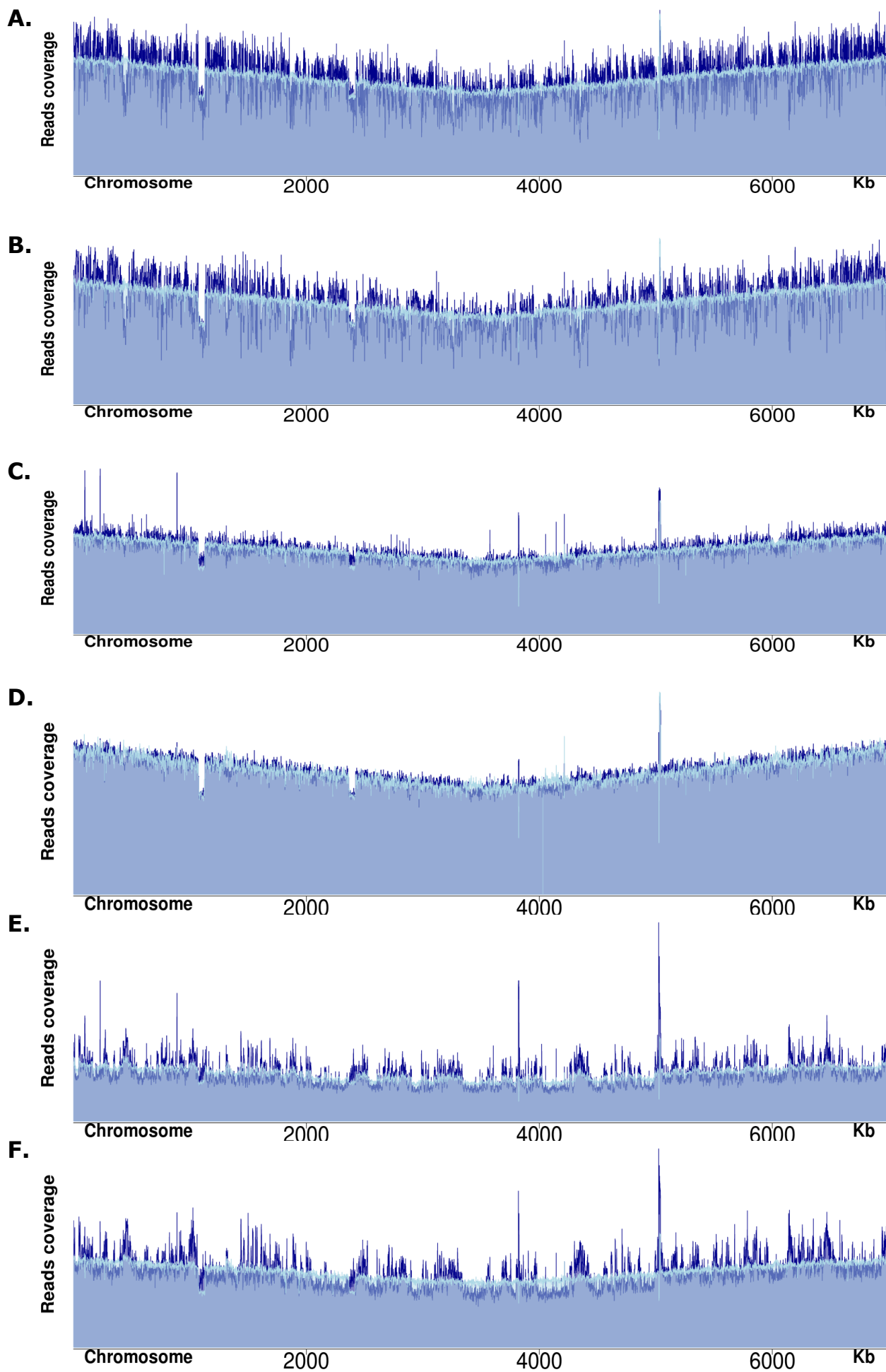

Fig. S4. Hołowka et al., 2017

Supplement: FIG S4 [file mbo006173577sf4.pdf]

**A.**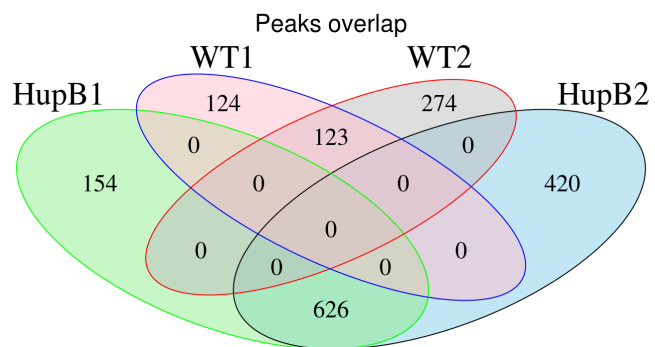**B.**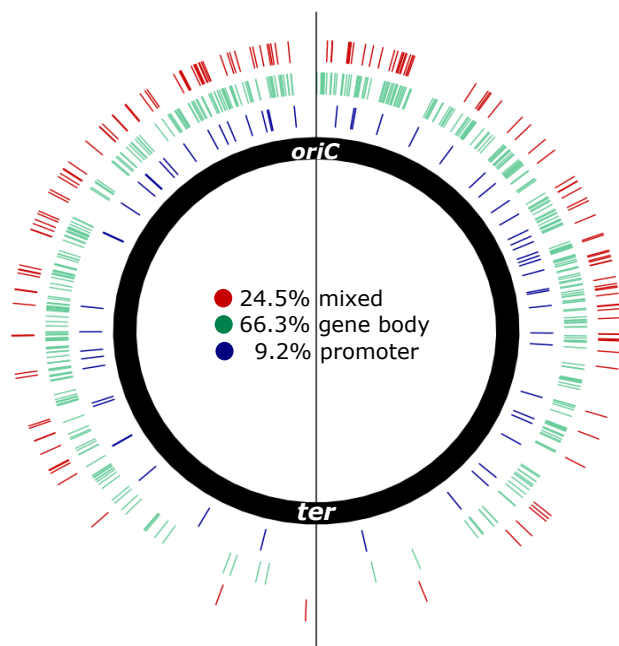**C.**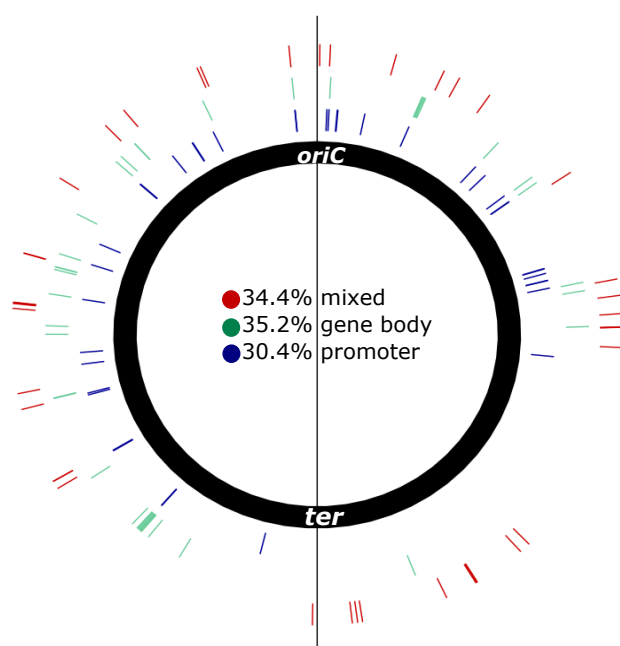

Fig. S5. Hołówka et al., 2017

Supplement: FIG S5 [file mbo006173577sf5.pdf]

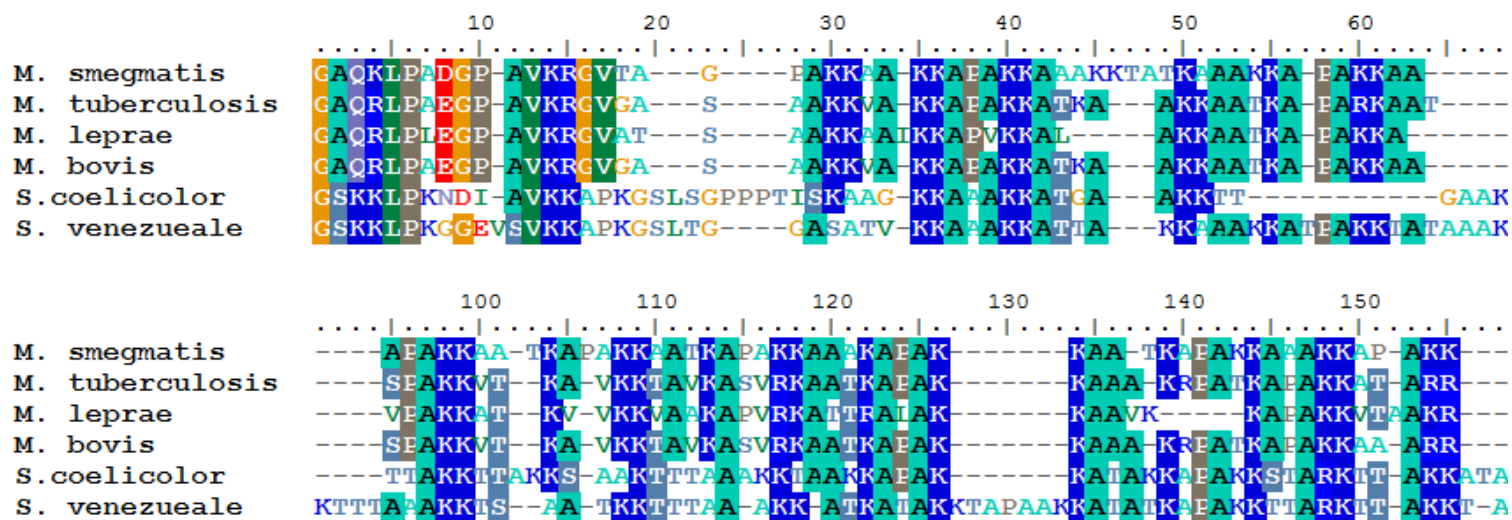

Fig. S6. Hołowka et al., 2017

Supplement: FIG S6 [file mbo006173577sf6.pdf]
