# Supplementary material for: HupB Is a Bacterial Nucleoid-Associated Protein with an Indispensable Eukaryotic-Like Tail
Source: mBio. 2017 Nov 7;8(6):e01272-17. doi: 10.1128/mBio.01272-17 (PMC5676037; doi:10.1128/mBio.01272-17)
Supplement: TABLE S1 [file mbo006173577st1.docx]

**Table S1. Oligonucleotides, plasmids and *M. smegmatis* mc^2^ 155 strains used in the study.**

| **Oligonucleotides** | | | |
| --- | --- | --- | --- |
| **Name** | | **Sequence 5’ to 3’** | **Tm (°C)** |
| Ms_2388_Fw_HindIII | | GGAAGCTTATGGAGGCTTTCACCACTC | 58 |
| Ms_2389_Rv_BamHI | | GGGGATCCCCTGCGGCCCTTCTTGG | 58 |
| GFP_BamHI_F_66 | | GGGGATCCCTGCCGGGCCCGGAGCTG | 66 |
| GFP_KpnI_R_64 | | CCGGTACCTTACTTGTACAGCTCGTCCATG | 64 |
| Ms_HupB2_Fw_KpnI | | CCGGTACCGCCAAGAAGGGCCGCAG | 58 |
| Ms_HupB2_Rv_PacI | | CCTTAATTAACTTGTGATGCCGGTGGAC | 58 |
| Ms_2389_5’_Rv_KpnI | | CCGGTACCCCAAAACCTCCGAAACCAG | 58 |
| Ms_2389_N_Rv_KpnI | | GGGGTACCTAAGCCAGAGATAACCGCCTT | 62 |
| Ms_p_MS_hupB_Fw_HindIII | | CCAAGCTTTCCGGGCCTTCGGCGACC | 64 |
| Ms_hupB_KpnI_Rv | | CCGGTACCTTACCTGCGGCCCTTCTTGG | 64 |
| Ms_2389_N_Rv_BamHI | | GGGGATCCGCCAGAGATAACCGCCTT | 56 |
| FLAGx3_BamHI/KpnI_Fw | | GATCCGACTACAAGGACGATGACGACAAGGACTACAAGGACGATGACGACAAGGACTACAAGGACGATGACGACAAGTGAGGTAC | 78 |
| FLAGx3_BamHI/KpnI_Rv | | CTCACTTGTCGTCATCGTCCTTGTAGTCCTTGTCGTCATCGTCCTTGTAGTCCTTGTCGTCATCGTCCTTGTAGTCG | 77 |
| PAmcherry_BamHI_Fw | | GGGGATCCTCGGCTGGCTCCGCTGCTG | 66 |
| KK_hupB_SalI_Fw | | AATTCGAAGCTTATCGATGTCGATCGTCCGGCCTGGAAACC | 60 |
| KK_(hupB)_paCh_Fw | | CCAAGAAGGGCCGCAGGTCGGCTGGCTCCGCTGCT | 62 |
| KK_(paCh)_hupB_Rv | | AGCAGCGGAGCCAGCCGACCTGCGGCCCTTCTTGG | 58 |
| KK_SalI_paCh_Rv | | GTACGCTAGTTAACTACGTCGATTACTTGTACAGCTCGTCCATG | 64 |
| **Plasmids** | | | |
| **Name** | | **Plasmid features** | **Reference** |
| pGoal17 | | ampicillin resistance, *oriE*, selective PacI cassete with *lacZ*, *sacB* and *kanR* genes | (1) |
| p2NIL | | kanamycin resistance, *oriE*, suicide vector for allelic replacement | (1) |
| p2NIL-HupB-EGFP-pGoal | | kanamycin resistance, *oriE*, *hupB-egfp* fusion gene, PacI cassette | this study |
| p2NIL-HupB-mCherry-pGoal | | kanamycin resistance, *oriE*, *hupB-mcherry* fusion gene, PacI cassete | this study |
| p2NIL-HupB_∆CTD_-EGFP-pGoal | | kanamycin resistance, *oriE*, *hupB_∆CTD_-egfp* fusion gene, PacI cassete | this study |
| p2NIL-HupB-FLAG-pGoal | | kanamycin resistance, *oriE*, *hupB-flag* fussion gene, PacI cassete | this study |
| p2NIL-HupB_∆CTD_-FLAG -pGoal | | kanamycin resistance, *oriE*, *hupB_∆CTD_ -flag* fusion gene, PacI cassete | this study |
| p2NIL-∆HupB-pGoal | | kanamycin resistance, *oriE*, PacI cassete | this study |
| p2NIL-HupB_∆CTD_ -pGoal | | kanamycin resistance, *oriE*, *hupB_∆CTD_* gene fragment, PacI cassete | this study |
| p2NIL-HupB_∆CTD_ | | kanamycin resistance, *oriE*, *hupB_∆CTD_* gene fragment, PacI cassete | this study |
| p2NIL-HupB_∆CTD_-PAmcherry | | kanamycin resistance, *oriE*, *hupB_∆CTD-_PAmcherry* fusion gene, PacI cassete | this study |
| pMV306 | | kanamycin resistance, *oriE*, *oriM*, *attP*, *attB* integrative vector for mycobacterial transformation | (2) |
| pMV-HupB | | kanamycin resistance, *oriE*, *attP*, *hupB* gene under natural promoter | this study |
| pMV-HupB*-*PAmcherry | | kanamycin resistance, *oriE*, *attP*, *hupB-PAmcherry* fusion gene under natural promoter | this study |
| pMV-HupB_∆CTD_*-*PAmcherry | | kanamycin resistance, *oriE*, *attP*, *hupB_∆CTD_-PAmcherry* fusion gene under natural promoter | this study |
| **Strains** | | | |
| **Name** | **Relevant genotype** | | **Source** |
| WT | *M. smegmatis* mc^2^ 155 | | laboratory stock |
| HupB-EGFP | *M. smegmatis* mc^2^155 *hupB-egfp* | | this study |
| HupB-mCherry | *M. smegmatis* mc^2^155 *hupB-mcherry* | | this study |
| HupB_∆CTD_ -EGFP | *M. smegmatis* mc^2^155 *hupB_∆CTD_ -egfp* | | this study |
| HupB-FLAG | *M. smegmatis* mc^2^155 *hupB-flag* | | this study |
| HupB_∆CTD_-FLAG | *M. smegmatis* mc^2^155 *hupB_∆CTD_ -flag* | | this study |
| ∆*hupB* | *M. smegmatis* mc^2^155 ∆*hupB* | | this study |
| HupB_∆CTD_ | *M. smegmatis* mc^2^155 *hupB_∆CTD_* | | this study |
| ∆*hupB*/p_NAT_*hupB* | *M. smegmatis* mc^2^155 ∆*hupB attB*L5::pMV306p_NAT_*hupB* | | this study |
| HupB-PAmCherry | *M. smegmatis* mc^2^155 ∆*hupB attB*L5::pMV306p_NAT_*hupB-PAmcherry* | | this study |
| HupB_∆CTD_-PAmCherry | *M. smegmatis* mc^2^155 ∆*hupB attB*L5::pMV306p_NAT_*hupB_∆CTD_-PAmcherry* | | this study |

**References**

1. Parish T, Stoker NG. 2000. Use of a flexible cassette method to generate a double unmarked Mycobacterium tuberculosis tlyA plcABC mutant by gene replacement. Microbiol Read Engl 146 ( Pt 8):1969–1975.

2. Triccas JA, Parish T, Britton WJ, Gicquel B. 1998. An inducible expression system permitting the efficient purification of a recombinant antigen from Mycobacterium smegmatis. FEMS Microbiol Lett 167:151–156.
